# BCL10-CARD11 Fusion Mimics an Active CARD11 Seed That Triggers Constitutive BCL10 Oligomerization and Lymphocyte Activation

**DOI:** 10.3389/fimmu.2018.02695

**Published:** 2018-11-20

**Authors:** Thomas Seeholzer, Susanne Kurz, Florian Schlauderer, Simone Woods, Torben Gehring, Simon Widmann, Katja Lammens, Daniel Krappmann

**Affiliations:** ^1^Research Unit Cellular Signal Integration, Institute of Molecular Toxicology and Pharmacology, Helmholtz Zentrum München - German Research Center for Environmental Health, Neuherberg, Germany; ^2^Gene Center, Ludwig-Maximilians University, Munich, Germany

**Keywords:** lymphocyte signaling, CARMA1–BCL10–MALT1 (CBM) signalosome complex, CARD11, NF- kappa B, MALT1 paracaspase

## Abstract

Assembly of the CARD11/CARMA1-BCL10-MALT1 (CBM) signaling complex upon T or B cell antigen receptor (TCR or BCR) engagement drives lymphocyte activation. Recruitment of pre-assembled BCL10-MALT1 complexes to CARD11 fosters activation of the MALT1 protease and canonical NF-**κ**B signaling. Structural data and *in vitro* assays have suggested that CARD11 acts as a seed that nucleates the assembly of BCL10 filaments, but the relevance of these findings for CBM complex assembly in cells remains unresolved. To uncouple cellular CARD11 recruitment of BCL10 and BCL10 filament assembly, we generated a BCL10-CARD11 fusion protein that links the C-terminus of BCL10 to the N-terminus of CARD11. When stably expressed in CARD11 KO Jurkat T cells, the BCL10-CARD11 fusion induced constitutive MALT1 activation. Furthermore, in CARD11 KO BJAB B cells, BCL10-CARD11 promoted constitutive NF-**κ**B activation to a similar extent as CARD11 containing oncogenic driver mutations. Using structure-guided destructive mutations in the CARD11-BCL10 (CARD11 R35A) or BCL10-BCL10 (BCL10 R42E) interfaces, we demonstrate that chronic activation by the BCL10-CARD11 fusion protein was independent of the CARD11 CARD. However, activation strictly relied upon the ability of the BCL10 CARD to form oligomers. Thus, by combining distinct CARD mutations in the context of constitutively active BCL10-CARD11 fusion proteins, we provide evidence that BCL10-MALT1 recruitment to CARD11 and BCL10 oligomerization are interconnected processes, which bridge the CARD11 seed to downstream pathways in lymphocytes.

## Introduction

Assembly of the CARD11/CARMA1-BCL10-MALT1 (CBM) signalosome channels T and B cell antigen-receptor (TCR/BCR) ligation to MALT1 protease activation and canonical NF-**κ**B signaling (1, 2). CARD11 phosphorylation, primarily in the central linker region, following antigenic stimulation induces conformational changes that expose the N-terminal CARD (Caspase Recruitment Domain) to recruit pre-assembled BCL10-MALT1 complexes (3, 4). Oncogenic CARD11 variants have been identified mainly in the coiled-coil domain, and these activating mutations promote chronic CBM assembly and NF-**κ**B-driven survival in diffuse large B cell lymphomas (DLBCL) in the absence of antigenic stimulation (5, 6).

It is well established that BCL10 associates with the CARD-containing scaffold protein CARD11 through heterotypic CARD-CARD interactions (7, 8). Overexpression studies indicate that BCL10, via its N-terminal CARD, forms filament-like clusters in cells, which are required for proper activation of canonical NF-**κ**B signaling (9). Aggregation of BCL10 in foci was also observed following TCR ligation in T cells (10). More recent *in vitro* structural studies, combined with molecular modeling, have demonstrated that the CARD of CARD11 can function as a seed to nucleate the assembly of BCL10 CARD filaments (11–13). *In vitro* BCL10 filaments can also form in the absence of CARD11, but CARD11 decreases the lag period of BCL10 polymerization and thus appears to function as an initiator of the process (11). Impaired MALT1 activity and NF-**κ**B signaling upon overexpression of CARD11 or BCL10 mutants, targeting either the heterotypic CARD11-BCL10 or the homotypic BCL10-BCL10 CARD interfaces, highlights the importance of the different CARD surfaces (12, 13). These experiments, however, did not address the contribution of the different interfaces to antigenic activation when expressed at endogenous levels. We have demonstrated that BCL10 oligomerization is also required for its recruitment to CARD11, indicating that both processes are highly interconnected (14). Thus, the cellular relevance of the CARD11 seeding function for BCL10 filament formation, as well as, the order of events after antigenic stimulation, have not been resolved.

Here, we used CRISPR/Cas9 technology to generate CARD11 and BCL10 KO T and B cell lines and stable lentiviral reconstitution, to investigate the cellular necessity of the CARD11 seed and BCL10 filaments in a clean genetic setup under physiological conditions. As noted earlier, we have been unable to definitively determine whether CARD11 nucleates BCL10 filaments or, if BCL10 filament formation happens prior to, or at the same time as CARD11 recruitment in stimulated T cells using missense mutations in the putative CARD11-BCL10 or BCL10-BCL10 interfaces alone (14). Thus, we uncoupled these processes by fusing BCL10 to CARD11 to bypass inducible recruitment and thereby were able to investigate the cellular necessity of CARD11 seeding and BCL10 oligomerization.

## Results

### BCL10 recruitment to CARD11 and BCL10 filament assembly are interconnected processes

**Figure 1:**
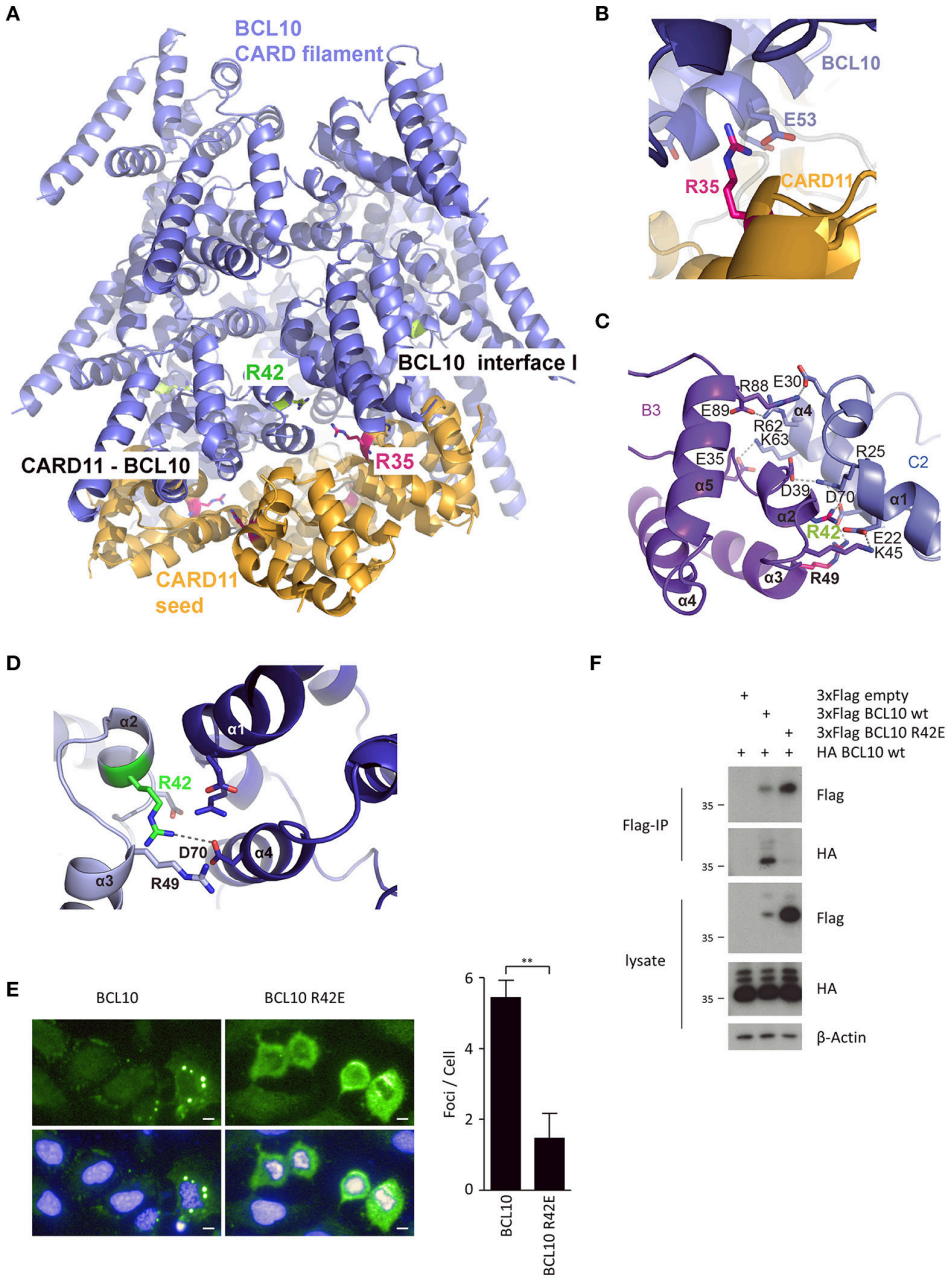
CARD-CARD interfaces in the CARD11-BCL10 structure. **(A)** Model of the CARD11-BCL10 filament structure. The oligomeric CARD11 seed (PDB 4LWD, orange) induces BCL10 (PDB 6GK2, blue) filament assembly through the heterotypic CARD11-BCL10 interface. **(B)** Close-up view of CARD11 R35 (magenta) contacting BCL10 E53 in the modeled CARD11-BCL10 interface. **(C,D)** Close-up view of the BCL10-BCL10 interface I. Residues involved in homotypic CARD-CARD association as observed in the cryo-EM structure (EMD-0013, PDB 6GK2) (14) are shown as sticks. The mutated residue R42 is highlighted in green. **(E)** Cellular distribution of BCL10 in U2OS cells was determined after transfection of BCL10-FS or BCL10 R42E-FS and aggregate clustering was detected by indirect confocal immunofluorescence microscopy. Scale bars depict 10 μm. Average number of aggregated foci was quantified by blinded counting >30 cells per condition (mean ± s.e.m.; **p ≤ 0.01). **(F)** HEK 293 cells were transfected with Flag- and HA-tagged BCL10 constructs as indicated, and self-association of HA-BCL10 WT to Flag-BCL10 WT or R42E mutant was determined after Flag-IP.

In order to predict mutations that would selectively interfere with CARD11 seed function or BCL10 self-assembly, we used structural modeling to fit the CARD11 seed onto the structure of BCL10 CARD filaments (Figure 1A). Therefore, the CARD11 CARD domain crystal structure was superimposed on to three BCL10 CARD domains at the bottom of the BCL10 filament cryo EM structure [Figure 1A; (13, 14)]. Since the structure of the CARD11-BCL10 interface has not been determined, the CARD11 BCL10 CARD/CARD interaction was modeled in consideration of the surface charge complementarity analysis of the BCL10-MALT1 filament cryo-EM structure and the crystal structure of the CARD11 (Supplementary Figures 1A–C). As noted earlier, distinct interfaces between the CARDs are required to mediate heterotypic CARD11-BCL10 interactions or homotypic BCL10-BCL10 interactions (12, 13). On the CARD11 side, R35 serves as a critical contact point to multiple residues in BCL10 including E53 and mediates recruitment of BCL10 to CARD11 [Figure 1B; (12)]. On BCL10, R42 contributes to the association at the BCL10-BCL10 interface I that controls BCL10 oligomerization (Figures 1C,D). The structure reveals that R42 is not predicted to confer CARD11-BCL10 interaction (11, 13, 14). *In vitro*, the BCL10 CARD mutation R42E prevents oligomerization of the BCL10-MALT1 complex (14). We expressed BCL10 WT and R42E in adherent U2OS cells to monitor formation of cellular clusters. Indeed, whereas overexpressed BCL10 WT forms cytosolic aggregates in U2OS cells, BCL10 R42E fails to cluster, indicating that oligomerization and filament formation is prevented by the mutation (Figure 1E). We performed co-immunoprecipitation (IP) of HA-BCL10 WT, together with Flag-BCL10 WT or R42E in HEK293 cells (Figure 1F). Despite the higher expression of BCL10 R42E, only Flag-BCL10 WT co-precipitated with HA-BCL10, validating that the mutation abolishes BCL10 self-association.

To rigorously test the function of CARD11 as a molecular seed and BCL10 as a filament forming CARD11 adaptor in B and T cells, we generated CARD11 and BCL10 KO Jurkat T cells, as well as CARD11 KO BJAB B cells, by CRISPR/Cas9 technology (Supplementary Figure 2). CARD11 KO Jurkat T and BJAB B cells were generated using sgRNA targeting Exon3, which induces double stranded breaks and frame shift mutations due to non-homologous end-joining (NHEJ) repair (Supplementary Figure 2A). An exon1-intron1 deletion strategy using two sgRNA was employed to knockout BCL10 from Jurkat T cells (Supplementary Figure 2B). Using these approaches, we obtained several clones that displayed loss of CARD11 or BCL10 expression as determined by Western Blot analysis (Supplementary Figures 2C–E). Destructive frame shift mutations in CARD11 or deletions of BCL10 in both alleles were confirmed by sequencing of the genomic loci in the respective KO cell clones (data not shown). We used PMA/Ionomycin (P/I) stimulation, which bypasses upstream TCR or BCR signaling by directly activating PKCθ or PKCβ in T and B cells and increasing cytosolic calcium levels. In line with the key role of the CBM complex, CARD11 or BCL10 deficiency abolished P/I-induced NF-**κ**B signaling in Jurkat T or BJAB B cells as evident from lack of IκBα degradation and NF-**κ**B DNA binding (Supplementary Figures 2C–E). In contrast, CBM-independent, TNFα-driven NF-**κ**B signaling as well as ERK activation were not affected by the absence of CARD11 in Jurkat T cells, demonstrating that loss of the CBM complex selectively affects antigenic signaling. Thus, the absence of CARD11 or BCL10 in Jurkat and BJAB cells faithfully mirrors the signaling defects observed in primary lymphocytes from CARD11^−/−^ or BCL10^−/−^ mice (15–17).

**Figure 2:**
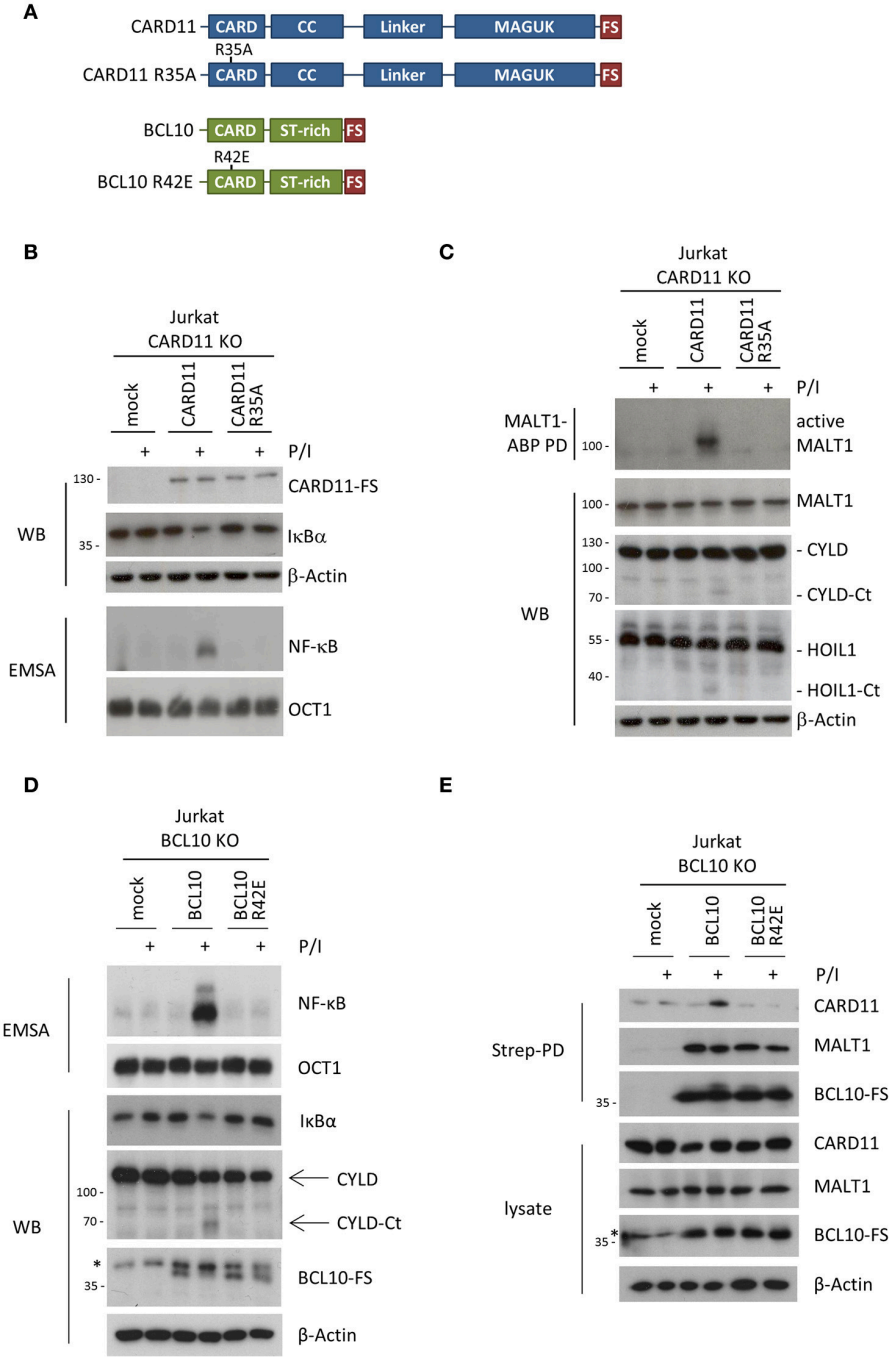
Effects of destructive heterotypic and homotypic CARD-CARD interface mutants. **(A)** Schematic presentation of CARD11 and BCL10 WT proteins and the respective CARD mutants R35A and R42E. **(B)** CARD11 KO Jurkat T cells were reconstituted with CARD11 WT or R35A. Cells were stimulated with P/I (30 min) and effects on NF-**κ**B signaling were determined by following IκBα degradation by WB and NF-**κ**B activation by EMSA. **(C)** CARD11 KO Jurkat T cells were reconstituted as in **(B)**, stimulated with P/I (30 min), and MALT1 protease activity was determined via MALT1-ABP PD assay and assessment of substrate cleavage (CYLD and HOIL1) by WB. **(D)** BCL10 KO Jurkat T cells were reconstituted with BCL10 or BCL10 R42E. Cells were stimulated with P/I (30 min) and NF-**κ**B signaling was analyzed as in **(B)**. MALT1 protease activity was determined by CYLD substrate cleavage by WB. **(E)** Recruitment of BCL10 or BCL10 R42E to CARD11 after P/I stimulation (15 min) in Jurkat T cells was monitored by ST-PD and subsequent WB. The asterisks indicate an unspecific band in the BCL10 WB.

We then performed rescue experiments in Jurkat KO T cells to study the molecular functions of the CARD11 and BCL10 CARDs, by introducing missense mutations (CARD11 R35A and BCL10 R42E) that are both predicted to selectively interfere with heterotypic CARD11-BCL10 and homotypic BCL10-BCL10 interactions, respectively [Figures 1, 2A; (12, 14)]. Jurkat CARD11 or BCL10 KO T cells were reconstituted by lentiviral transduction and comparable infection rates for epitope-tagged (FS: Flag-StrepTag2) CARD11 and BCL10 constructs were obtained, as determined by co-expression of the surface marker ΔCD2 (Supplementary Figures 3A,B). Equivalent expression of WT and mutant CARD11 or BCL10 proteins at close to endogenous levels was confirmed by Western Blotting (Supplementary Figures 3A,B). Functionally, lack of CARD11-dependent NF-**κ**B activation after P/I stimulation in CARD11 KO cells was rescued upon reconstitution with CARD11 WT, but not with the CARD11 R35A mutant (Figure 2B). We also assessed activation of the MALT1 protease by substrate cleavage (CYLD and HOIL-1) (18) and labeling of active MALT1 by a biotinylated MALT1 activity-based probe (ABP) followed by biotin pull-down (PD) to capture active MALT1 [Figure 2C; (19)]. Again, CARD11 WT, but not R35A, could rescue P/I stimulated MALT1 protease activation in Jurkat T cells, revealing that this mutation abolishes all CARD11 downstream function. On the side of BCL10, we confirmed that rescue of BCL10 WT, but not BCL10 R42E, was able to trigger MALT1 activation and mediate NF-**κ**B downstream signaling in response to P/I stimulation [Figure 2D; (14)]. Since the structural analyses suggested that BCL10 R42E would selectively disrupt the BCL10-BCL10 but not the CARD11-BCL10 interface (13), we asked if BCL10 R42E could still be recruited to CARD11 upon stimulation. However, no stimulation-dependent binding to CARD11 was detected with BCL10 R42E mutant [Figure 2E; (14)]. The data demonstrate that CARD11 recruitment and BCL10 filament formation are interconnected processes. Thus, destructive mutations in the CARD11-BCL10 or BCL10-BCL10 interfaces alone are unable to resolve whether CARD11 acts as a seed to induce BCL10 oligomerization or if an initial BCL10 filament assembly may be required for the recruitment of BCL10-MALT1 to CARD11.

### BCL10-CARD11 fusion drives constitutive MALT1 activation through BCL10 oligomerization in jurkat T cells

**Figure 3:**
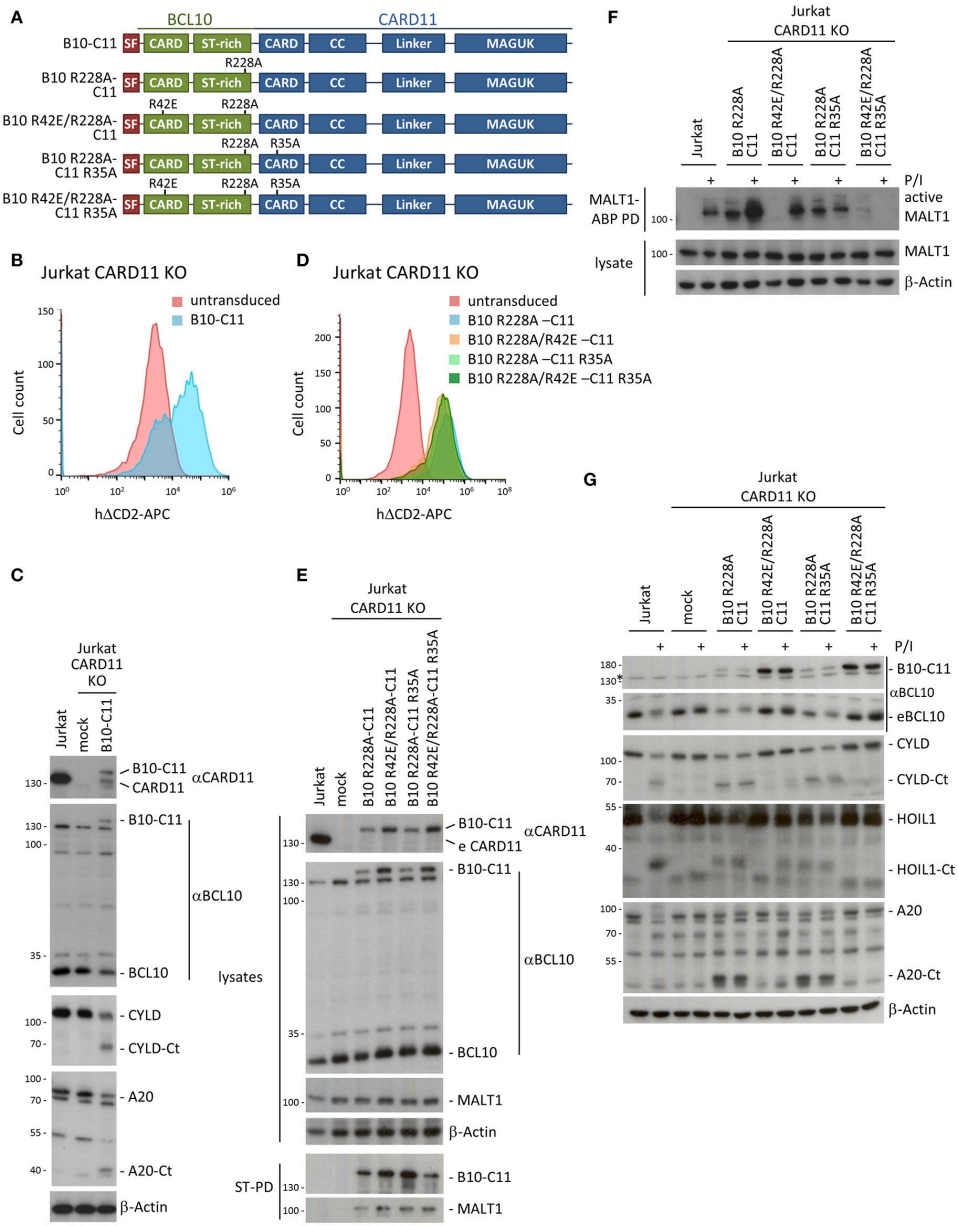
A chimeric BCL10-CARD11 fusion protein induces constitutive MALT1 protease activity in Jurkat T cells through the BCL10 oligomerization interface. **(A)** Schematic representation of BCL10-CARD11 (B10-C11) fusion proteins with the respective CARD mutants. **(B)** Transduction efficiency of CARD11 KO Jurkat T cells with BCL10-CARD11 (B10-C11) was analyzed by FACS using the surface marker ΔCD2. **(C)** Protein expression of B10-C11 fusion construct and cleavage of MALT1 substrates compared to mock and parental Jurkat T cells was analyzed by WB. **(D)** Transduction efficiency of CARD11 KO Jurkat T cells with different B10 R228A-C11 fusion constructs as in **(B)**. **(E)** Expression of the different B10 R228A-C11 fusion proteins compared to mock and parental Jurkat T cells was analyzed by WB and binding to MALT1 assessed by Strep-PD. **(F)** CARD11 KO Jurkat T cells were reconstituted with B10 R228A-C11 fusion proteins as indicated. Active MALT1 prior or after P/I stimulation (30 min) was detected in the extracts with biotin-labeled MALT1-ABP and MALT1-ABP PD followed by WB. **(G)** CARD11 KO Jurkat T cells were reconstituted and treated as in **(F)** and cleavage of MALT1 substrates (CYLD, HOIL1, A20) was detected by WB.

To test the necessity for BCL10 self-association downstream of CARD11, we designed a system that bypasses inducible CARD11-BCL10 association. For this we cloned chimeric proteins that covalently fuse BCL10 through its C-terminus to the N-terminus of CARD11 (Figure 3A). We lentivirally transduced CARD11 KO Jurkat T cells with the SF-tagged BCL10-CARD11 construct (herein referred to as B10-C11 fusion) (Figure 3B). We detected a faint but distinct band corresponding to the expected size of the B10-C11 fusion using αBCL10 and αCARD11 antibodies (Figure 3C). Notably, expression of the B10-C11 protein triggered constitutive cleavage of CYLD and A20, as well as a reduction in endogenous BCL10, which are all MALT1 substrates (18). However, the BCL10-CARD11 fusion itself, which contains a MALT1 cleavage site at R228 in the BCL10 moiety (20), was also prone to processing, giving rise to a fragment the size of endogenous CARD11 (Figure 3C). Thus, the data clearly indicate that fusion of BCL10 to CARD11 is sufficient to induce MALT1 activation.

To avoid the indirect reconstitution of CARD11 from the cleaved B10-C11 fusion, we designed all further BCL10-CARD11 fusion constructs so that they contained the MALT1 cleavage resistant BCL10 R228A mutation (Figure 3A). The fusion constructs include the destructive mutations R42E in the BCL10 CARD, and R35A in the CARD11 CARD, both alone and in combination. All B10-C11 fusion constructs were transduced into CARD11 KO Jurkat T cells, yielding equivalent infection efficiencies as judged by ΔCD2 expression (Figure 3D). The chimeric proteins were expressed below the level of endogenous CARD11 and moreover, the fusions containing a functional WT BCL10 CARD were consistently expressed at lower levels compared to the BCL10 mutants R42E that prevent BCL10 oligomerization (Figure 3E). StrepTactin pull-downs (ST-PD) demonstrated that the BCL10-CARD11 fusion proteins retained the ability to bind endogenous MALT1, excluding that the fusion or point mutations in the CARDs interfere with MALT1 recruitment (Figure 3E).

Using biotin-PD after incubation with bio-MALT1-ABP, we tested MALT1 protease activity in extracts of untreated and P/I stimulated Jurkat T cells (Figure 3F). Expression of B10 R228A-C11, containing two functional CARDs, as well as the B10 R228A-C11 R35A fusion, with an inactivation only in the CARD11 CARD, induced strong MALT1 protease activity that was equivalent to the activation obtained in Jurkat T cells after P/I stimulation. Despite robust MALT1 activation, the R228A exchange in BCL10 prevented cleavage of the fusion constructs and thus the appearance of CARD11 (Figure 3E). Constitutive MALT1 activation was abrogated in the B10-C11 fusion proteins carrying the oligomerization-defective BCL10 R42E mutation, providing evidence that constitutive MALT1 activation is driven through oligomerization of endogenous BCL10 via the BCL10 CARD (Figure 3F). Interestingly, MALT1 activity was further enhanced after stimulation of B10 R228A-C11 expressing cells and this strictly relied on the CARD11 CARD, because the R35A mutation prevented stimulation-dependent induction. In line, even though the B10 R42E/R228A-C11 fusion containing an intact CARD11 CARD was unable to induce constitutive MALT1 activation, it was still able to mediate MALT1 activation in P/I-stimulated Jurkat T cells. Thus, stimulation dependent activation seems to rely on the recruitment of endogenous BCL10 to the CARD11 CARD in the context of the BCL10-CARD11 fusion protein. In fact, the B10 R42E/R228A-C11 fusion acted completely independently of the BCL10 CARD and exactly mirrored the rescue observed when using CARD11 WT (see Figures 2B,C). In agreement with these data, the triple mutant B10 R42E/R228A-C11 R35A neither promoted constitutive, nor rescued, stimulus-dependent MALT1 activation.

To confirm these findings on the level of MALT1 substrates, we assessed cleavage of CYLD, HOIL1 and A20 in B10-C11 expressing cells (Figure 3G). Cleavage of the three substrates was observed in Jurkat T cells expressing the fusions with an intact BCL10 CARD (B10 R228A-C11 and B10 R228A-C11 R35A), confirming that constitutive MALT1 protease activity relies on BCL10 oligomerization. The intact CARD11 CARD in B10 R42E R228A-C11 still conferred inducible substrate cleavage, which was especially evident for HOIL1 that is also most strongly cleaved after P/I stimulation of Jurkat T cells. Again, destruction of both CARDs in B10 R42E/R228A-C11 R35A led to complete loss of constitutive and inducible MALT1 activation. Thus, covalent attachment of BCL10 to the N-terminus of CARD11 is sufficient to induce MALT1 protease activation, which still relies on the oligomerization interface of the fused BCL10 moiety. These data support the concept that, in cells, CARD11 acts as a seed to induce BCL10 filament assembly.

### Transient expression of BCL10-CARD11 induces NF-**κ**B activation in jurkat T cells

**Figure 4:**
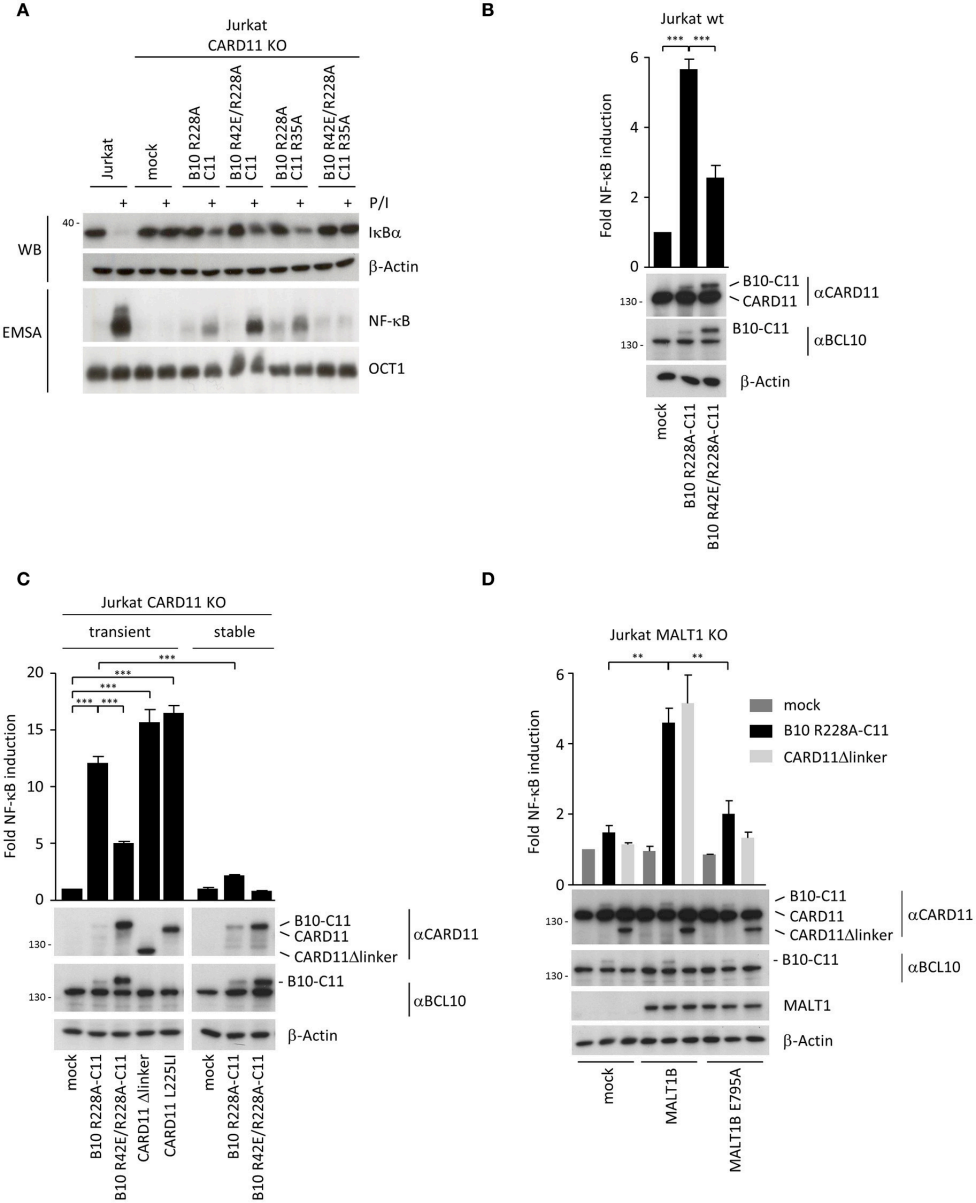
Transient expression of chimeric BCL10-CARD11 fusion protein in Jurkat T cells triggers NF-**κ**B activation. **(A)** NF-**κ**B signaling was analyzed in CARD11 KO Jurkat T cells stably reconstituted with different B10 R228A-C11 fusion proteins as indicated. IκBα degradation was determined by WB and NF-**κ**B activation was analyzed by EMSA. **(B)** B10-C11 fusion constructs were transiently expressed in Jurkat T cells together with a dual NF-**κ**B reporter, and NF-**κ**B induction was assessed by a dual luciferase reporter assay. Protein levels were analyzed by WB. Results are displayed relative to mock-transfected cells (mean ± s.e.m.; *n* = 5). **(C)** Jurkat CARD11 KO cells were transiently or stably reconstituted with B10-C11 fusion or CARD11 constructs and NF-**κ**B activity determined as in **(B)**. Expression of the fusion constructs was verified by WB (mean ± s.e.m.; *n* = 3). **(D)** Transient expression of B10 R228A-C11 and CARD11 Δlinker in Jurkat MALT1 KO cells stably reconstituted with mock, MALT1 IsoB wildtype and the TRAF6 binding motif mutant E795A, respectively. NF-**κ**B activity was determined as described in **(B)** and protein levels visualized on WB (mean ± s.e.m.; *n* = 3). **p ≤ 0.01, ***p ≤ 0.001.

Interestingly, when we tested activation of NF-**κ**B by B10-C11 fusion constructs in CARD11 or BCL10 KO Jurkat T cells we noticed a severely blunted response in EMSA (Figure 4A). There was a weak induction of constitutive NF-**κ**B DNA binding in B10 R228A-C11, but IκBα degradation and NF-**κ**B activation was only mildly triggered after P/I stimulation, revealing that stable expression of the active BCL10-CARD11 fusion may promote a stage of unresponsiveness in Jurkat T cells. Again, missense mutations in both CARDs completely prevented constitutive, as well as, inducible NF-**κ**B activation in the context of the B10-C11 fusion protein. We switched to a transient transfection system and NF-**κ**B reporter assays to investigate if BCL10-CARD11 fusion proteins can activate NF-**κ**B. Indeed, NF-**κ**B was strongly induced by the expression of B10 R228A-C11 fusion activated in parental Jurkat T cells or CARD11 KO Jurkat T cells (Figures 4B,C). However, in line with the EMSA results overall NF-**κ**B activation was strongly diminished when the reporter assay was performed in CARD11 KO Jurkat T cells that stably express the B10-C11 fusion constructs (Figure 4C). Further, the oligomerization-deficient BCL10 R42E mutant severely reduced NF-**κ**B activation by the BCL10-CARD11 fusion protein. Again, also in transient transfection we observed that BCL10-CARD11 was expressed at much lower levels compared to the B10 R42E/R228A-C11 protein, suggesting that there is a counter-selection against the expression of the active BCL10-CARD11 fusion. NF-**κ**B induction of BCL10-CARD11 fusion protein was comparable to the induction achieved by oncogenic CARD11 L225LI or CARD11 Δlinker, especially taking into account the much weaker expression of the fusion protein [Figure 4C; (6, 21)]. To check if the generated BCL10-CARD11 fusion construct does not trigger unphysiological NF-**κ**B that bypasses the necessity of known regulators, we determined the requirement for MALT1 and for TRAF6 recruitment to MALT1 (Figure 4D). NF-**κ**B activation in response to antigenic stimulation is abolished MALT1 KO Jurkat T cells and signaling can be rescued by transduction of MALT1A or MALT1B, but not the respective MALT1 TRAF6 binding mutants (22). As expected, expression of B10 R228A-C11 or CARD11 Δlinker was unable to activate NF-**κ**B in MALT1 KO Jurkat T cell (Figure 4D). While NF-**κ**B activity was recovered by viral complementation with MALT1B WT, the MALT1B E795A mutant that destroys the only functional TRAF6 binding motif on MALT1B failed to rescue reporter gene expression, proving that MALT1 and TRAF6 are utilized by the BCL10-CARD11 fusion protein to activate NF-**κ**B [Figure 4D; (22–24)]. Thus, while transient expression of the BCL10-CARD11 fusion protein promotes NF-**κ**B activation, NF-**κ**B responses are dampened in Jurkat T cells after stable expression of the fusion proteins. To corroborate whether the BCL10-CARD11 fusion protein can also compensate for BCL10 deficiency, we transduced BCL10 KO Jurkat T cells with the B10 R228A-C11 fusion protein (Supplementary Figure 4A). Indeed, fusion of BCL10 to CARD11 was able to trigger constitutive CYLD, A20, and HOIL1 cleavage and thus to drive MALT1 protease activation in BCL10 deficient cells (Supplementary Figure 4B). Again, mutation of BCL10 and CARD11 CARD (B10 R42E/R228A-C11 R35A) prevented constitutive and inducible MALT1 activation, underscoring that dimerization/oligomerization of the fusion proteins is required. Similar to the situation in CARD11 KO cells, NF-**κ**B activation was blunted upon stable expression of B10 R228A-C11 in BCL10 KO Jurkat T cells.

### BCL10-CARD11 fusion acts like oncogenic CARD11 in BJAB B cells

**Figure 5:**
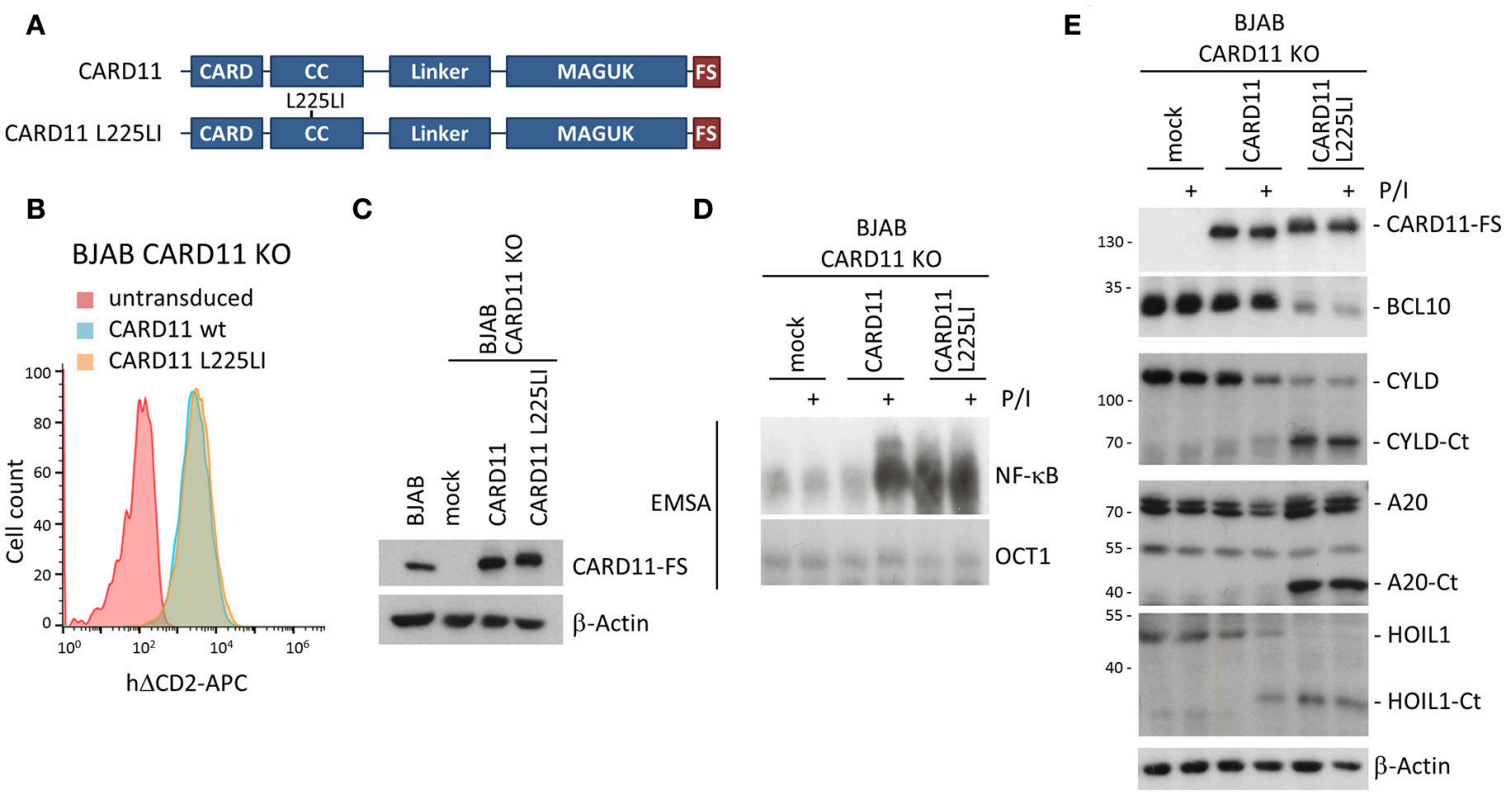
Rescue of CARD11 KO BJAB B cells by CARD11 WT and oncogenic CARD11 L225LI. **(A)** Schematic representation of CARD11 and CARD11 L225LI proteins. **(B)** Transduction of CARD11 KO BJAB B cells with CARD11 or CARD11 L225LI expressing lentiviruses was analyzed by the surface marker ΔCD2 using FACS. **(C)** CARD11 and CARD11 L225LI protein expression, compared to parental BJAB B cells, was determined by WB. **(D)** CARD11 KO BJAB B cells after reconstitution with CARD11 WT or oncogenic CARD11 L225LI were analyzed for NF-**κ**B activation after P/I stimulation (30 min) by EMSA. **(E)** MALT1 protease activity upon P/I stimulation (30 min) in CARD11 KO BJAB B cells reconstituted with CARD11 or CARD11 L225LI was determined by CYLD, A20, and HOIL1 cleavage on WB.

To better explore the impact of stable BCL10-CARD11 fusion on NF-**κ**B activation, we switched to GCB DLBCL derived BJAB B cells, which under basal conditions are devoid of NF-**κ**B activity, but overexpression of oncogenic CARD11 mutants induces strong chronic NF-**κ**B activity (5, 6, 25). We generated CARD11 KO BJAB B cells (Supplementary Figure 2D) and confirmed that the phenotype of the BJAB KO cells was caused by loss of CARD11. For this we reconstituted the cells with CARD11 WT, or the oncogenic CARD11 coiled-coil (CC) mutant L225LI that induces robust NF-**κ**B and proliferation upon overexpression in B cells [Figure 5A; (25, 26)]. After viral transduction, BJAB B cells expressed CARD11 WT and CARD11 L225LI slightly above endogenous levels (Figures 5B,C). CARD11 WT was able to recover P/I-inducible NF-**κ**B and MALT1 protease activation, but was not associated with constitutive activation (Figures 5D,E). In contrast, transduction of CARD11 L225LI was sufficient to promote constitutive NF-**κ**B activation, as well as cleavage of the MALT1 substrates BCL10, CYLD, A20 and HOIL1. This effect was not further augmented by P/I stimulation (Figures 5D,E). Thus, CARD11 KO BJAB B cells represent a valid system to elucidate the impact of BCL10-CARD11 fusions on NF-**κ**B signaling.

**Figure 6:**
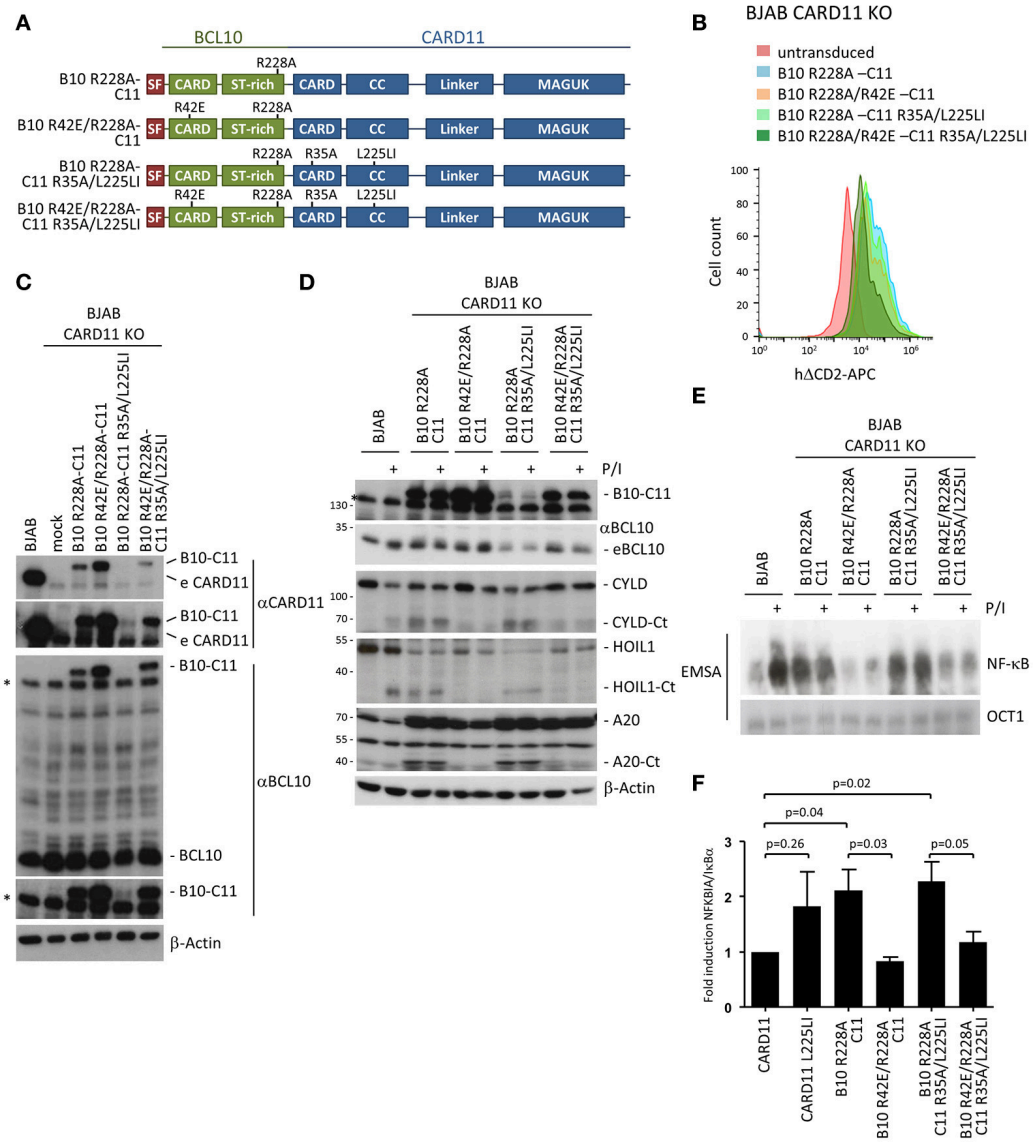
Constitutive NF-**κ**B and MALT1 activation by an oncogenic BCL10-CARD11 fusion protein relies on the BCL10 CARD interface in BJAB B cells. **(A)** Schematic representation of BCL10-CARD11 (B10-C11) fusion proteins containing CARD and oncogenic coiled-coil (CC) mutations. **(B)** Transduction of CARD11 KO BJAB B cells with B10 R228A-C11 fusion constructs, as depicted, was analyzed for the ΔCD2 surface marker by FACS. **(C)** Protein expression of the fusion constructs compared to parental BJAB B cells was determined by WB. **(D)** CARD11 KO BJAB B cells were reconstituted with B10 R228A-C11 fusion proteins as indicated. Constitutive and P/I-inducible (30 min) MALT1 activity was detected by CYLD, A20, and HOIL1 cleavage. **(E)** CARD11 KO BJAB B cells were reconstituted as in **(D)** and NF-**κ**B activity was analyzed by EMSA. **(F)** Expression of the NF-**κ**B target gene NFKBIA/IκBα in CARD11 and B10 R228A-C11 expressing BJAB B cells was determined by quantitative RT-PCR. All values were normalized to the housekeeping gene RPII and related to CARD11 (mean ± s.e.m, *p*-values as indicated).

Next, we expressed B10 R228A-C11 and B10 R42E/R228A-C11 in CARD11 KO BJAB B cells (Figure 6A). In addition, we sought to determine whether an oncogenic mutant of CARD11 also relies on BCL10 oligomerization. We have previously shown that the activating potential of CARD11 L225LI is abolished by the R35A mutation (25). Therefore, we expressed the B10-C11 L225LI fusion constructs in the context of the CARD destructive R35A mutation so that they should rely on the BCL10 CARD (B10 R228A-C11 R35A/L225LI and B10 R42E/R228A-C11 R35A/L225LI) (Figure 6A). All constructs were transduced in BJAB B cells to a similar extent, as determined by the surface marker ΔCD2, but protein expression was far below endogenous CARD11 (Figures 6B,C). As observed in Jurkat T cells, B10 R228A-C11 constructs containing an intact BCL10 CARD were expressed at much lower levels and the variant containing the oncogenic mutation (B10 R228A-C11 R35A/L225LI) was hardly detectable (Figure 6C lane 5). However, just like in Jurkat T cells, the B10 R228A-C11 fusion induced strong constitutive MALT1 activation, as evident from CYLD, HOIL1 and A20 cleavage, which strictly relied upon the BCL10 CARD interface (Figure 6D). Furthermore, the BCL10-CARD11 fusion combined with the oncogenic mutation L225LI (B10 R228A-C11 R35A/L225LI) induced MALT1 activation, despite its very low expression in the BJAB B cells. Again, constitutive MALT1 activation in the oncogenic BCL10-CARD11 fusion required BCL10 oligomerization, but was independent of the CARD11 CARD (Figure 6D).

Further, the fusion of BCL10 to CARD11 was sufficient to induce constitutive NF-**κ**B activation in the BJAB B cells that was not further enhanced by P/I treatment (Figure 6E). Constitutive NF-**κ**B activation was equivalent to that induced by the oncogenic CARD11 variant L225LI (Figure 5D). Despite its very weak expression, the oncogenic fusion construct B10 R228A-C11 R35A/L225LI also induced strong NF-**κ**B activation, suggesting that the combination of fusion with the oncogenic mutant can generate a super-activator that is capable of further boosting CBM signaling. The weak expression of the B10 R228A-C11 R35A/L225LI suggests that there is a strong counter-selection against expression of the hyper-active fusion protein. Again, the constitutive activation observed with expression of the fusion constructs was solely driven by the BCL10 filament interface and was severely reduced in cells expressing the double CARD mutant B10 R42E/R228A-C11 R35A/L225LI. Finally, we confirmed that the fusion constructs also induce NF-**κ**B-dependent gene expression by demonstrating that the prototype NF-**κ**B target gene, NFKBIA/IκBα, is upregulated to a similar degree in the BJAB B cells expressing either CARD11 L225LI or the activating B10-C11 fusion constructs (Figure 6F). Thus, with respect to signaling, the fusion of BCL10 to CARD11 acts like an oncogenic CARD11 variant in B cells, through oligomerization of the BCL10 CARD interface.

## Discussion

BCL10 CARD filaments are formed *in vitro* and the molecular architecture of these clusters has been elucidated by cryo-electron microscopy (11, 13, 14). Although it is possible for BCL10 filaments to form in the absence of CARD11, the CARD of CARD11 appears to promote the initiation of BCL10 clustering in a cell free system. Further, CARD11 is solely located at the tip of the BCL10 filaments, lending support to the hypothesis that CARD11 functions as the molecular seed for BCL10 oligomerization (11, 13). Structure-guided missense mutations in the CARDs of CARD11 or BCL10 have been generated to show that destruction of putatively homotypic (BCL10-BCL10) or heterotypic (CARD11-BCL10) CARD-CARD interactions impedes NF-**κ**B signaling and MALT1 protease activation after overexpression in cells (13, 14).

The functional impact of missense mutations in the CARDs of CARD11 or BCL10 on stimulus-dependent CBM complex signaling has not been thoroughly investigated. By reconstituting CARD11 or BCL10 KO Jurkat T cells, we assessed how destruction of putative CARD11-BCL10 or BCL10-BCL10 interfaces would affect signaling when expressed at endogenous levels. As expected, the CARD11 mutation R35A prevented T cell stimulation due to its inability to recruit BCL10 (12, 25). In addition, the BCL10-BCL10 interface mutant R42E could not rescue NF-**κ**B signaling in BCL10 KO cells, which confirms the critical function of BCL10-BCL10 interface I for filament assembly and signaling (13). Recently, we have been able to solve the architecture of the BCL10-MALT1 filaments using cryo-EM (14). Surprisingly, we found that different mutations in the interface I that lead to loss of BCL10 filament assembly also abolished CARD11 recruitment of BCL10 and thus CBM complex formation (14). Thus, our results question if CARD11 acts as the seed to nucleate BCL10 filaments, or whether an initial oligomerization of BCL10 is required for the recruitment to CARD11 in cells. Alternatively, our data would also be in line with a model in which CARD11-BCL10 association, but not BCL10 oligomerization, is critical to promote signaling in lymphocytes, and that filament formation is only observed with recombinant BCL10 or after high overexpression of BCL10.

To clarify the necessity of the CARD11 seed and BCL10 filaments for T and B cell activation, we fused BCL10 to CARD11 to bypass the initial step of heterotypic CARD-CARD interaction between CARD11 and BCL10. Even though this is an artificial system, it provided the first opportunity to examine the contribution of the individual CARDs in CARD11 and BCL10. Remarkably, stable expression of the BCL10-CARD11 fusion protein promoted strong and chronic MALT1 protease activity in Jurkat T cells. While NF-**κ**B activation was blunted in transduced Jurkat T cells, the transient transfection of BCL10-CARD11 activated NF-**κ**B to a similar extent as an oncogenic CARD11 variant or CARD11 lacking the negative regulatory linker region (CARD11 Δlinker) (5, 6, 21). Interestingly, the BCL10-CARD11 fusion is not prone to auto-inhibition by the CARD11 linker, which acts as an inhibitory domain (27). In the fusion protein the BCL10 CARD is exposed and most likely not accessible for the CARD11 linker, especially when considering that an additional CARD cannot be bound by the linker in a 1:1 stoichiometry. Moreover, CARD11 and BCL10 CARDs are homologous, but also quite distinct and the BCL10 CARD may complex with the CARD11 linker, which would be critical for auto-inhibition (5, 27). Despite the disrupted auto-inhibition, the BCL10-CARD11 fusion protein signals to NF-**κ**B via MALT1 and the TRAF6 binding motif on MALT1, underscoring that it utilizes the same mechanisms as a physiological CBM complex following T cell activation.

Despite the structural elucidation of BCL10-MALT1 filaments *in vitro*, the nature and relevance of cellular BCL10 oligomeric structures has not been fully resolved. BCL10 tends to aggregate via its CARD into oligomeric clusters and extended filaments after overexpression in cells (7, 9). Endogenous BCL10 forms oligomeric structures in antigen-stimulated T cells termed POLKADOTS (“punctuated and oligomeric killing or activating domains transducing signals”), which are cellular foci that serve as functional platforms for recruiting NF-**κ**B signaling mediators following TCR stimulation (10, 28). Size exclusion chromatography demonstrated that in stimulated Jurkat T cells or ABC DLBCL tumor cells CARMA1, BCL10, and MALT1 assemble into higher order complexes with an apparent molecular weight >1 Mio. Da and the purified CBM complex displayed a filament-like structure in electron microscopy (13, 29). However, by increasing the concentration of BCL10 during the process of CBM purification, BCL10 filament formation may be initiated *in vitro* rather than taking place in living cells. Thus, there is good evidence that BCL10 can cluster via the CARD in cells, but the existence of large helical BCL10 filaments under physiological conditions in antigen-stimulated T or B cells has not been formally demonstrated. Since it may be difficult to imagine that the BCL10-CARD11 fusion itself can form long filaments in the absence of endogenous BCL10, activation by the fusion in BCL10 KO cells may indicate that BCL10-dependent dimerization or short oligomerization may be sufficient for lymphocyte activation. As for endogenous CARD11-associated BCL10, monitoring of BCL10 filament formation in the context of BCL10-CARD11 fusion is difficult with current methods, because of low expression levels. High resolution imaging techniques will be necessary to solve the extent of oligomerization and the cellular architecture of the BCL10 clusters. However, so far imaging in lymphocytes has been hampered by the unavailability of high quality antibodies as well as the small and round-shaped T and B cells that contain very little cytoplasm.

An important question that remains is the *in vivo* necessity for the formation of large, extended BCL10 filaments for signal propagation. The lack of NF-**κ**B and MALT1 activation in BCL10 R42E in the context of the BCL10-CARD11 fusion reflects the need for BCL10 dimerization/oligomerization, but does not prove the requirement for higher order filaments. Alternatively, BCL10-MALT1 recruitment to CARD11 and BCL10 dimerization or short oligomers may be sufficient to facilitate cellular processes such as ubiquitination of CBM complex components. A number of ubiquitin ligases [e.g., TRAF6, LUBAC (linear ubiquitin chain assembly complex) and cIAP2] are recruited to the CBM complex and conjugation of mono-ubiquitin or poly-ubiquitin chains of different topology on all subunits has been implicated in triggering MALT1 activation and/or downstream signaling (1, 2). The complexity of ubiquitin-dependent regulation is exemplified by BCL10. K48-, K63-, or M1-linked ubiquitin chains are primarily conjugated on K17, K31, and/or K63 in the BCL10 CARD (30–32). Ubiquitination of these lysine residues is required for NF-**κ**B activation in T cells and pro-survival signaling in ABC DLBCL cells (31–33). Deficiency in HOIP/RNF31, the catalytic subunit of LUBAC, suppresses NF-**κ**B signaling in Jurkat T cells whereas HOIP activating mutations enhance NF-**κ**B-dependent pro-survival in ABC DLBCL cells, supporting that M1-linked ubiquitin chains on BCL10 are enhancing CBM complex signaling (32, 34). However, at the same time BCL10 is degraded by lysosomal or proteasomal pathways and these processes are controlled by at least partially overlapping ubiquitination sites (31, 35–37). In addition, K48-linked poly-ubiquitination of CARD11 induces its degradation (38). Thus, the BCL10-CARD11 fusion protein is most likely strongly affected by regulatory ubiquitination and ubiquitin conjugation in each moiety may be responsible for activation, as well as the high turnover of the active BCL10-CARD11 fusions. Due to the multi-layered ubiquitin regulation and the uneven expression levels of active vs. inactive BCL10-CARD11 fusions, this system is not an ideal tool to study the relevance of BCL10 ubiquitination. However, it will be important to clarify in how far BCL10 oligomerization and potential filament formation cooperate with ubiquitination processes to induce downstream signaling or alternatively, if BCL10 ubiquitination may also facilitate CBM complex assembly.

In BJAB B cells stable BCL10-CARD11 expression activates MALT1 and NF-**κ**B as strong as oncogenic variants of CARD11 derived from DLBCL tumor patients (5, 6, 25, 26), clearly showing that the proximity of BCL10 and CARD11 alone is sufficient to activate downstream signaling pathways. Thus, the BCL10-CARD11 fusion already provides compelling evidence that CARD11 acts as a seed in cells to boost lymphocyte activation. By introducing point mutations we were also now able to unravel the contribution of the two different CARD interfaces. Constitutive activity of the BCL10-CARD11 fusion no longer required the presence of an intact CARD11 CARD, but was solely driven by the BCL10 oligomerization interface. In contrast, stimulus-dependent MALT1 activation of the BCL10-CARD11 fusion protein relied on an intact CARD11 CARD, which most likely as in CARD11 WT serves as a platform for recruitment and oligomerization of endogenous BCL10. Interestingly, introduction of the active oncogenic mutation L225LI into the CARD11 coiled-coil (CC) region in the context of the BCL10-CARD11 resulted in a fusion protein that was hyper-active, despite the very low expression level in the BJAB B cells. Indeed, all active BCL10-CARD11 fusion proteins were expressed at a lower level compared to their inactive counterparts. However, the very low expression of the hyper-active fusion constructs indicates that there is a strong counter-selection against the expression of these constitutively active variants in T and B cells. Since all fusions constitutively activate the MALT1 paracaspase in the two cell types, it is possible that the sustained MALT1 cleavage of mRNA processing factors Regnase-1 and Roquin1/2 may exert toxic effects that impair T and B cell survival (39, 40). More work is needed to understand under which circumstances chronic activation induces cell survival or toxicity.

While expression of BCL10-CARD11 fusion drives NF-**κ**B activation in BJAB B cells, constitutive and inducible NF-**κ**B signaling is severely impaired in Jurkat T cells expressing the fusion protein. Even though NF-**κ**B activation is impaired in Jurkat T cells, MALT1 protease is still strongly activated by BCL10-CARD11 fusions, demonstrating that not all CBM downstream effects are affected. Further, the reduced responsiveness relies on the stable expression of the BCL10-CARD11 fusion protein and it needs to be explored what cell-intrinsic mechanisms operate in Jurkat T cells that counteract constitutive activation of the canonical NF-**κ**B pathway downstream of the CBM complex. Of note, T lymphocytes from patients with germline activating CARD11 mutations are anergic, while the B cells are activated and expanding, causing a phenotype called BENTA (B cell expansion with NF-**κ**B and T cell anergy) (41, 42). It will be interesting to see whether similar negative regulatory mechanisms observed in Jurkat T cells driven by an active BCL10-CARD11 fusion could also operate in primary T cells.

Our data suggest that upon the initial BCL10 recruitment to CARD11, the weak heterotypic interaction of the monomeric CARDs needs to be stabilized by further interactions arising from the oligomerized CARD11 seed and the helical BCL10 filaments. The additional contact points within this multimeric complex are essential for a high affinity binding and the formation of a stable CBM complex that is competent to trigger downstream signaling. Furthermore, the structural rigidity in the core BCL10-MALT1 filament may stabilize the binding, which is in line with the observation that CARD11 binding is also reduced when BCL10 is not complexed with MALT1 (14). In conclusion, we demonstrate that the recruitment of BCL10-MALT1 to CARD11 and BCL10-MALT1 filament formation are highly interconnected processes that cooperate to drive CBM downstream effects in response to physiological or pathological activation of T and B cells.

## Materials and methods

### Cell lines and treatments

Cell lines were maintained at 37°C in a humidified atmosphere at 5% CO_2_. Jurkat T cells and BJAB B cells were cultured in RPMI 1640 Medium, and U2OS and HEK293T cells in DMEM. Media were supplemented with 10% (Jurkat T cells, U2OS, HEK293T) or 15% (BJAB B cells) fetal calf serum, 100 U/ml penicillin and 100 μg/ml streptomycin. U2OS, HEK293T and BJAB B cells were obtained from the DSMZ, Jurkat T cells were authenticated by the Authentication Service of the Leibniz Institute DSMZ. Jurkat T cells were stimulated with Phorbol 12-Myristate 13-Acetate (PMA: 200 ng/ml; Merck) and Ionomycin (300 ng/ml; Calbiochem) for 30 min, except if otherwise stated.

### DNA constructs and antibodies

DNA constructs and antibodies used in this study are listed in Tables 1, 2, respectively.

**Table 1:** DNA constructs.

pHAGE-ΔCD2-T2A	Lentiviral transfer vector used (43)
pMD2.G	Lentiviral packaging construct (Addgene: Plasmid #12259)
psPAX2	Lentiviral packaging construct (Addgene: Plasmid #12260)
pHAGE-ΔCD2-T2A-SF	Lentiviral transfer vector (mock)
CARD11-FS constructs	CARD11, CARD11 R35A, and CARD11 L225LI in pHAGE-ΔCD2-T2A (25)
BCL10-FS constructs	BCL10 and BCL10 R42E in pHAGE-ΔCD2-T2A
SF-BCL10-CARD11 (B10-C11) constructs	CARD11 and CARD11 mutants (R35A, R35A/L225LI) in SF-BCL10, SF-BCL10 R42E, SF-BCL10 R228A, or SF-BCL10 R42E/R228A containing pHAGE-ΔCD2-T2A
pGL3-6xNF-**κ**B luc	NF-**κ**B reporter firefly luciferase (44)
pRL-TKluc	TK reporter renilla luciferase (Promega)

**Table 2:** Antibodies.

**Primary antibodies**	**Source**
A20/TNFAIP3 (D13H3)	Cell Signaling
BCL10 (C-17)	Santa Cruz
BCL10 (H-197)	Santa Cruz
CARMA1/CARD11 (1D12)	Cell Signaling
CYLD (E-10)	Santa Cruz
HOIL-1 (S150D)	MRC
IκBα (L35A5)	Cell Signaling
IκBα (phospho-Ser32/36) (5A5)	Cell Signaling
MALT1 (B-12)	Santa Cruz
StrepTagII	IBA
β-Actin (I-19)	Santa Cruz
anti-CD2-APC (RPA-2.10)	eBioscience
**Secondary antibodies**	**Source**
HRP-conjugated anti-goat	Jackson ImmunoResearch
HRP-conjugated anti-mouse	Jackson ImmunoResearch
HRP-conjugated anti-rabbit	Jackson ImmunoResearch
HRP-conjugated anti-sheep	Jackson ImmunoResearch
Alexa Fluor488-donkey anti-mouse	Invitrogen

### Generation and reconstitution of knock-out cells

Bicistronic expression vector px458 expressing Cas9 and sgRNA (45, 46) was digested with *BbsI* and the linearized vector was gel purified. Targeting oligos (CARD11: 5′CTCATCAATGACCTTACACTGACGCAGGTAGG 3′BCL10: 5′AGTGAGGTCCTCCTCGGTGA 3′and 5′TTCCGCTTTCGTCTCCCGCT 3′) for each targeting site positioned as depicted in Supplementary Figures 2A,B, were annealed and ligated to the linearized vector. Jurkat T cells or BJAB B cells (4–8 × 10^6^) were electroporated (220 V and 1,000 μF) using a Gene pulser X (Biorad) with px458 plasmids expressing sgRNA targeting CARD11 or BCL10, as well as a EGFP expression cassette. Twenty-four to forty-eight hours after electroporation, GFP positive cells were sorted using a MoFlow sorting system. Isolation of clonal cell lines was achieved by serial dilutions and was followed by an appropriate expansion period. KO cell clones were initially identified by detecting CARD11 or BCL10, respectively, by Western Blot. Clones lacking protein expression were genotyped by genomic PCR using intronic primers flanking targeting sides.

For reconstitution, lentivirus was produced in HEK293T cells. 1 × 10^6^ HEK293T cells were seeded in 8 ml DMEM medium (10% FCS, 1% Pen/Strep) in 10 cm^2^ dishes and grown overnight at 37°C. The next day, the cells were transfected with 1.5 μg of the packaging vector psPAX2, 1.0 μg of the lentiviral envelope plasmid pMD2.G and 2 μg pHAGE transfer vector using X-tremeGENE HP DNA Transfection Reagent (Roche) according to the manufacturer's protocol. After 3 days the supernatant of the HEK293T cells containing the virus was sterile filtered (0.45 μm). For transduction, virus supernatant was transferred to 5 × 10^5^ Jurkat T cells or BJAB B cells. For BJAB B cells, supernatant was concentrated with Amicon centrifugal filter units (100 K) prior to transduction. The solution was filled up with RPMI medium (10% FCS, 1% Pen/Strep) to a final volume of 2–2.5 ml and mixed with Polybrene (8 μg/ml). To enhance transduction efficiency, BJABs were centrifuged for 1 h at 500 × g. Forty to seventy-two hours later, cells were washed with PBS (without calcium and magnesium) and re-suspended in 1–2 ml RPMI medium (10% FCS, 1% Pen/Strep). Seven to ten days after transduction infection was analyzed by determining ΔCD2 surface expression by FACS and CARD11 or BCL10 protein expression by Western Blot. Only cells yielding a transduction efficiency of >90% as determined by FACS analysis, were used for further analyses.

Generation and reconstitution of MALT1-deficient Jurkat T cells has been described (22).

### Flow cytometry (FACS)

Surface expression of ΔCD2 after lentiviral transduction of BJAB B cells or Jurkat T cells was assessed by incubating 200 μl of the cell culture for 15 min at room temperature with 2 μl anti-CD2-APC (RPA-2.10) antibody. Cells were centrifuged (1,100 rpm, 5 min) and re-suspended in 250 μl PBS before FACS using Attune Acoustic Focusing Flow Cytometer.

### Cell lysis and precipitations

For analysis of expression via Western Blot or EMSA, cells (1–3 × 10^6^) were harvested (300 × g, 5 min, 4°C) and washed once with ice cold PBS. The pellet was resuspended in 80–100 μl high salt buffer (20 mM HEPES pH 7.9, 350 mM NaCl, 20% glycerol, 1 mM MgCl_2_, 0.5 mM EDTA, 0.1 mM EGTA, 1% NP-40, 1 mM DTT, 10 mM sodium fluoride, 8 mM β-glycerophosphate, 300 μM sodium vanadate and Roche protease inhibitor cocktail). For binding studies, cells (1–5 × 10^7^) were lysed in co-IP buffer (25 mM HEPES pH 7.5, 150 mM NaCl, 0.2% NP-40, 10% glycerol, 1 mM DTT, 10 mM sodium fluoride, 8 mM β-glycerophosphate, 300 μM sodium vanadate and protease inhibitor cocktail). Lysate controls were mixed with 4xSDS loading dye and boiled. For StrepTactin pull-downs (ST-PD), 20–40 μl Strep-Tactin Sepharose (1:1 suspension) was used for binding of Strep-tagged BCL10 or BCL10-CARD11 fusion overnight at 4°C rotating. Sepharose beads were pelleted after incubation (100 × g, 4 min, 4°C), washed 3x with co-IP buffer, and boiled after the addition of 20 μl 2xSDS loading dye (Roti-load). Lysates and ST-PDs were separated by SDS-PAGE and analyzed by Western Blot.

### Western blot

Proteins were transferred onto PVDF-membranes for immunodetection using an electrophoretic semi-dry transfer system. After transfer, membranes were blocked with 5% BSA for 1 h at RT and incubated with specific primary antibodies (indicated above, diluted 1:1,000 in 2.5% BSA/PBS-T) overnight at 4°C. Membranes were washed in PBS-T before the addition of HRP-coupled secondary antibodies (indicated above, 1:7,000 in 1.25% BSA in PBS-T; 1 h, RT). HRP was detected by enhanced chemiluminescence (ECL) using the LumiGlo reagent (Cell Signaling) according to manufacturer's instructions.

### Electrophoretic mobility shift assay (EMSA)

For EMSAs, double-stranded NF-**κ**B (H2K: fw: 5′-GATCCAGGGCTGGGGATTCCCCATCTCCACAGG-3′, rev: 5′- GATCCCTGTGGAGATGGGGAATCCCCAGCCCTG-3′), and OCT1 binding sequences (fw: 5′- GATCTGTCGAATGCAAATCACTAGAA-3′, rev: 5′-GATCTTCTAGTGATTTGCATTCGACA-3′) were labeled with [α-^32^P] dATP using Klenow Fragment (NEB). To monitor DNA binding, whole cell lysates (3–6 μg) were incubated for 30 min at RT with shift-buffer [20 mM HEPES pH 7.9, 120 mM KCl, 4% Ficoll, 5 mM DTT, 10 μg BSA and 2 μg poly-dI-dC (Roche)] and radioactive double stranded NF-**κ**B or OCT1 probes (10,000–20,000 cpm). Samples were separated on a 5% polyacrylamide gel in TBE buffer, vacuum-dried and exposed to autoradiography.

### Confocal immunofluorescence microscopy

The localization and distribution of BCL10 was analyzed by seeding U2OS cells in 96-well plates. Cells were transfected using Lipofectamine 3000 Transfection Reagent (Invitrogene) according to the manufacturer's instructions. To optimize cell attachment, CellCarrier-96 black plates (PerkinElmer) were coated with 100 μl poly-D-lysine at a concentration of 50 μg/ml. Twenty-four hours after transfection, cells were washed with PBS and fixed with 60 μl Methanol (−20°C) for 5–10 min at room temperature and then washed 3x with PBS. For immunostaining, cells were blocked in 2% BSA in PBS. Cells were incubated with primary antibody (anti-StrepTagII) in blocking buffer for 2 h at RT. Cells were washed 3x for 10 min at RT, before incubation with secondary antibody (Alexa Fluor488-donkey anti-mouse) in blocking buffer for 1 h at RT. Cell nuclei were visualized by incubation with Hoechst 33342 dye (Life Technologies) in PBS at a concentration of 0.5 μg/ml for 30 min at RT. Afterwards the cells were washed and covered with PBS, sealed with foil and kept at 4°C in the dark until microscopy. Confocal microscopy was performed with an Operetta high-content imaging system (Perkin-Elmer).

### Labeling and biotin pull-down (PD) of active MALT1

The biotin-labeled MALT1 activity based probe (MALT1-ABP) has been described previously (19). To investigate MALT1 protease activity, Jurkat T cells (3 x 10^7^) were washed with PBS, and lysed in 600 μl co-IP buffer without protease inhibitors for 30 min at 4°C. After clearing the lysates by centrifugation (20,000 × g, 4°C, 15 min), 30 μl were collected as lysate control, mixed with 4x SDS loading buffer and boiled for 5 min at 95°C. To 550 μl of the supernatant 12 μl High Capacity Streptavidin Beads (Thermo Fisher) was added and incubated for 1 h at 4°C for pre-clearing. The beads were pelleted (4,000 rpm, 2 min, 4°C) and 450 μl of supernatant was mixed with MALT1-ABP probe (0.1 μM final concentration). After 1 h rotating at room temperature, 15 μl High Capacity Streptavidin Beads was added before 1–2 h incubation at 4°C (rotating). Beads were collected and washed 3x with co-IP buffer without protease inhibitors. Beads were re-suspended in 22 μl 2x SDS loading buffer and boiled at 95°C for 7 min before SDS-PAGE and Western Blot analysis.

### NF-κB reporter assay

For NF-**κ**B luciferase reporter assays, 8 × 10^6^ Jurkat cells were transfected by electroporation with 2 μg NF-**κ**B firefly luciferase reporter plasmid, 1 μg renilla luciferase and 6 μg of CARD11 and BCL10-CARD11 fusion constructs using 220 V and 1,000 μF (Gene Pulser X, BioRad). After cultivation for 72 h cells were lysed in passive lysis buffer and luciferase activity measured using a dual luciferase reporter kit according to the manufacturer's protocol (Promega). All luciferase values were calculated in relation to the Renilla control.

### Quantitative reverse-transcriptase polymerase chain reaction (qRT-PCR)

RNA was isolated (QIAGEN RNeasy Kit) and equal amounts of RNA (InviTrap Spin Universal RNA Mini Kit, 1060100200, Stratec) were transcribed into cDNA using the Verso cDNA synthesis Kit (AB1453B, Thermo Fisher Scientific). Quantitative real-time (qRT) PCR was performed using KAPA SYBR FAST qPCR Master Mix (KAPA Biosystems) and standard LightCycler protocol on a Roche LightCycler 480. RNA Polymerase II (PolII) served as internal standard. The following primers were used: fw: 5′-CCGCACCTCCACTCCATCC-3′ rev: 5′-ACATCAGCACCCAAGGACACC-3′; RPII fw: 5′-GCACCACGTCCAATGACA-3′ rev: 5′-GTGCGGCTGCTTCCATAA-3′. Results represent the mean and standard error of the mean of three independent experiments.

### Structural model of CARD11-BCL10 and BCL10-BCL10 interaction surfaces

The CARD11-BCL10 CARD/CARD interaction model was prepared under consideration of the surface charge complementarity analysis of the BCL10-MALT1 filament cryo EM structure (EMD-0013, PDB 6GK2) and the crystal structure of the CARD11 domain (PDB 4LWD) (Supplementary Figure 1). The CARD11 CARD structure has been superimposed on the BCL10 CARD within the BCL10-MALT1 filament using the align command in PyMOL (The PyMOL Molecular Graphics System, Version 2.0 Schrödinger, LLC). Under consideration that the mutation of residue R35 in CARD11 abolishes the interaction to BCL10 (12), we propose that the mainly positively charged top of the CARD11 CARD interacts with the negatively charged bottom of the Bcl10 filament (Supplementary Figures 1B,C). The electrostatic surface was calculated with the program APBS Tool 2.1 implemented in PyMOL (47).

### Statistical analysis

Data for luciferase reporter assay, quantitative RT-PCR and fluorescence microscopy was analyzed for statistical significance using the unpaired Student's *t*-test (^**^p ≤ 0.01, ^***^p ≤ 0.001). Sample size (*n*) is specified for each experiment and data are shown as mean ± s.e.m.

## Author contributions

TS and SK conceived and performed most experiments, analyzed, and interpreted the data. FS and KL conceived and performed biophysical experiments and provided structural expertise. SW, TG, and SWi generated, verified and analyzed KO cells. DK conceived the study, experiments, wrote the manuscript, and secured funding. All authors read, acknowledged, and helped with the final version of the manuscript.

### Conflict of interest statement

The authors declare that the research was conducted in the absence of any commercial or financial relationships that could be construed as a potential conflict of interest.
