# Supplementary material for: BCL10-CARD11 Fusion Mimics an Active CARD11 Seed That Triggers Constitutive BCL10 Oligomerization and Lymphocyte Activation
Source: Front Immunol. 2018 Nov 20;9:2695. doi: 10.3389/fimmu.2018.02695 (PMC6255920; doi:10.3389/fimmu.2018.02695)
Supplement: Supplementary file 1 [file Data_Sheet_1.PDF]

SUPPLEMENTARY INFORMATION

Seeholzer et al.

SUPPLEMENTARY FIGURES AND LEGENDS

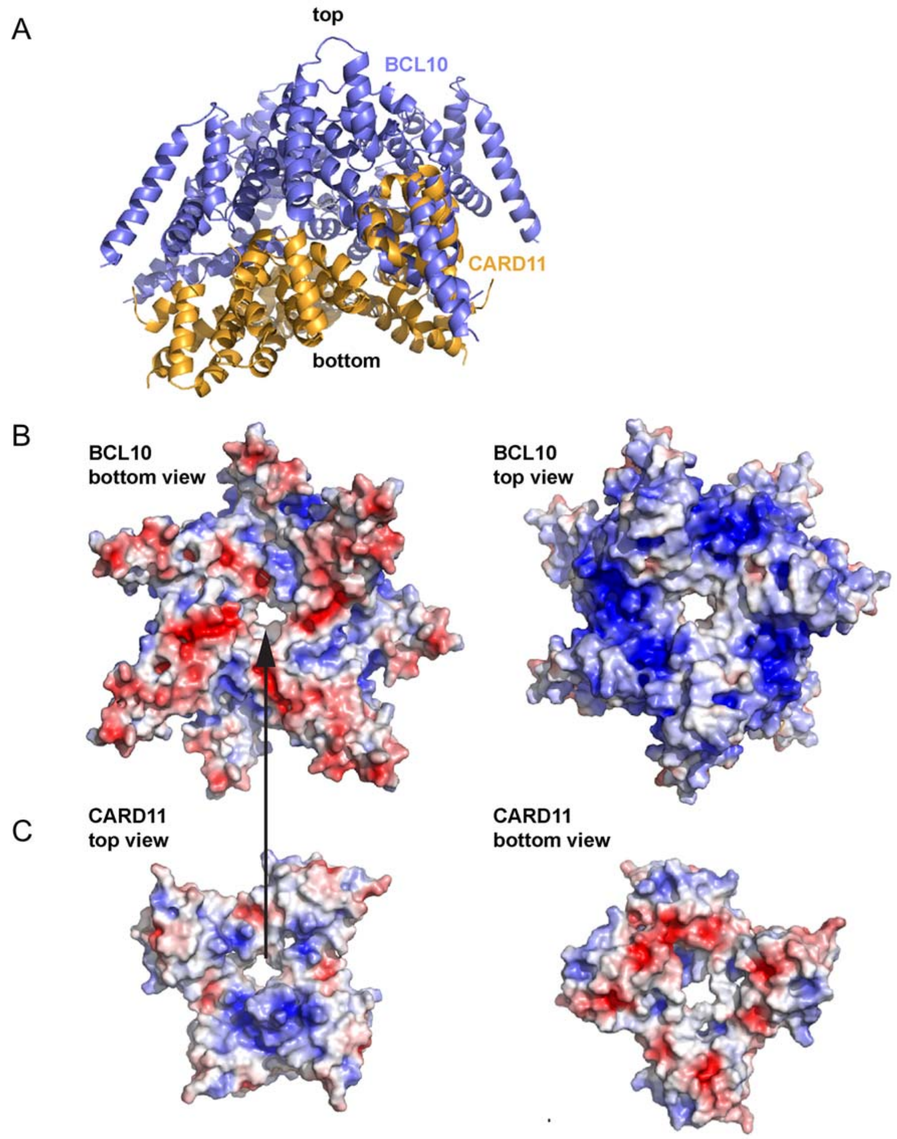

Supplementary Figure 1: Generation of the CARD11 BCL10 CARD domain interaction model. (A) Ribbon model of 5 BCL10 CARD domains (PDB 6GK2) arranged in the filament and colored blue shown together with the superimposed CARD11 CARD domains (PDB:4LWD) colored orange. (B-C) Electrostatic surface potential of the BCL10 filament calculated with APBS and shown from two

opposite views. The bottom of the BCL10 filament is mainly negatively charged (colored red), whereas the blue color of the top view indicates a positively charged surface. (C) Electrostatic surface potential of the CARD11 CARD domain seed model from top and bottom view, respectively. The surfaces indicate that the positively charged top of the CARD11 seed is charged complementary to the negatively charged bottom of the BCL10 filament.

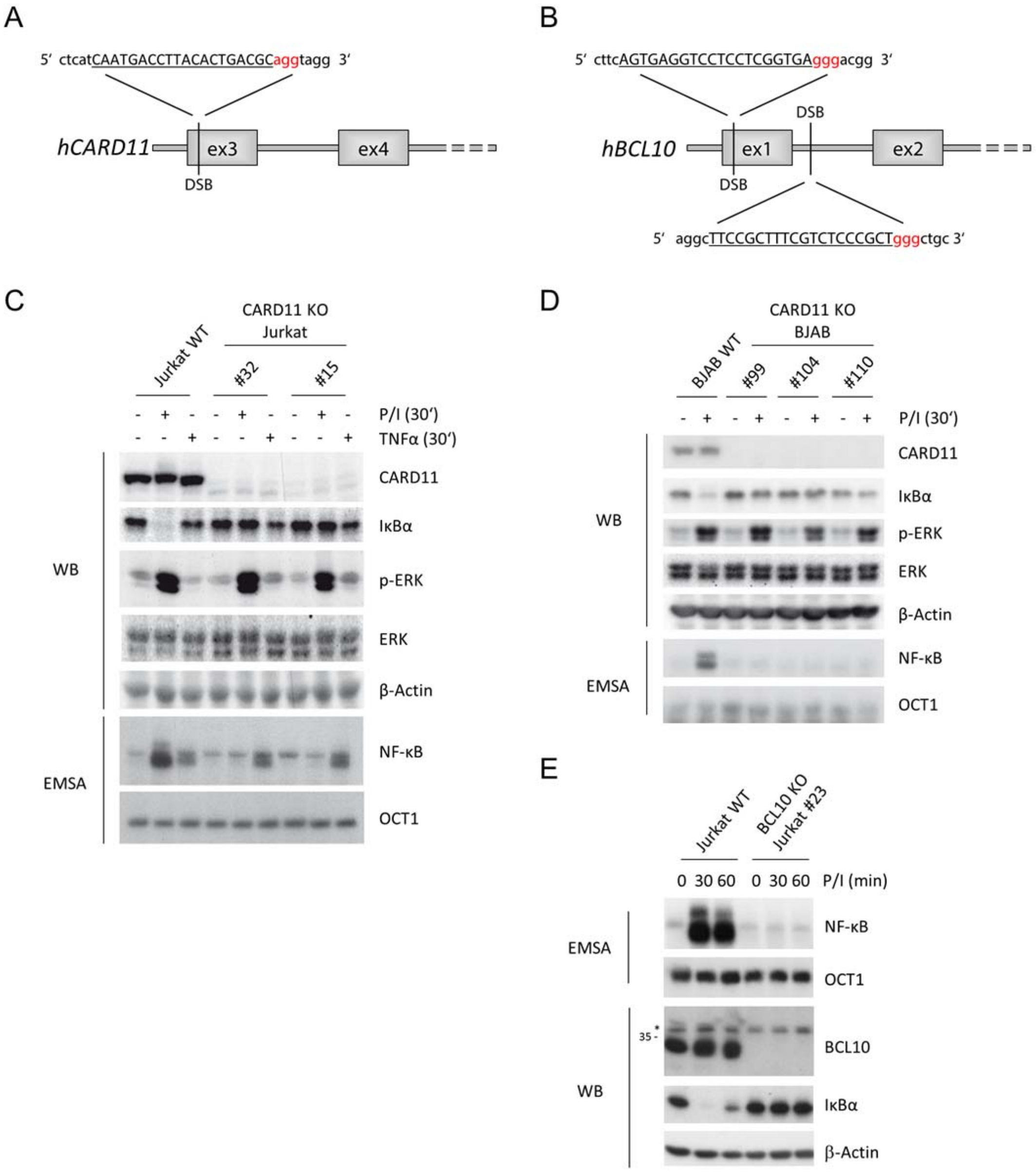

Supplementary Figure 2: Generation, verification and signaling in CARD11 and BCL10 KO cells. (A and B) Schematic of the Cas9/sgRNA-targeting sites in the *CARD11* gene (A) and *BCL10* gene (B). The sgRNA-targeting sequences are underlined and the protospacer-adjacent motif (PAM) is labeled in red. Induced double-strand breaks are marked with dotted lines. (C) Loss of CARD11 expression and CBM-driven P/I stimulation, but not CBM-independent TNF stimulation, in two independent CARD11 KO Jurkat T cell clones. Protein expression, IκBα degradation and phosphorylation of ERK were

analyzed by WB. NF-κB activation was determined by EMSA. (D) Loss of CARD11 expression and CBM-driven P/I stimulation in three independent CARD11 KO BJAB B cell clones. Protein expression, IκBα degradation and pERK were analyzed by WB. NF-κB activation was determined by EMSA. (E) Loss of BCL10 expression and CBM-driven P/I stimulation in a BCL10 KO Jurkat T cell clone. Protein expression and IκBα degradation was analyzed by WB. NF-κB activation was determined by EMSA.

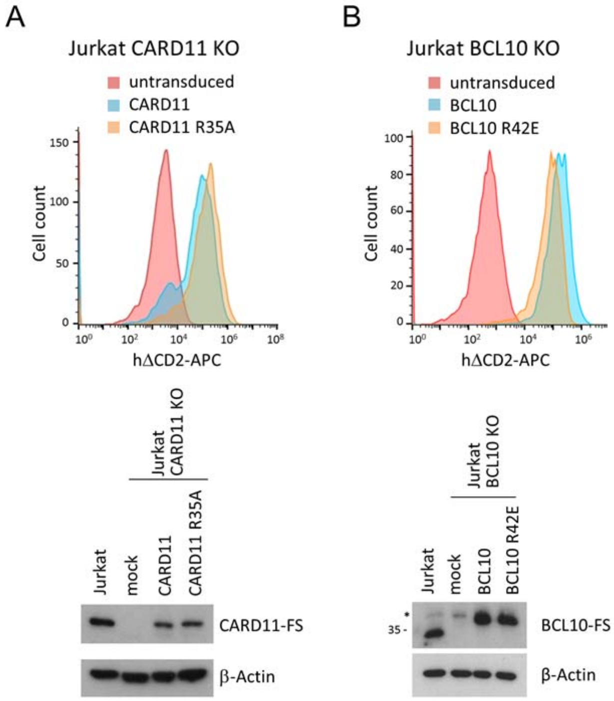

Supplementary Figure 3: Reconstitution of CARD11 and BCL10 KO Jurkat T cells. (A) Transduction of CARD11 KO Jurkat T cells with mock, CARD11 WT and CARD11 R35A expressing lentiviruses was analyzed by FACS (ΔCD2 surface marker) and protein expression by WB compared to parental Jurkat T cells. (B) Transduction of BCL10 KO Jurkat T cells with mock, BCL10 WT and BCL10 R42E expressing lentiviruses was analyzed as in A. The asterisk marks an unspecific band in the BCL10 WB.

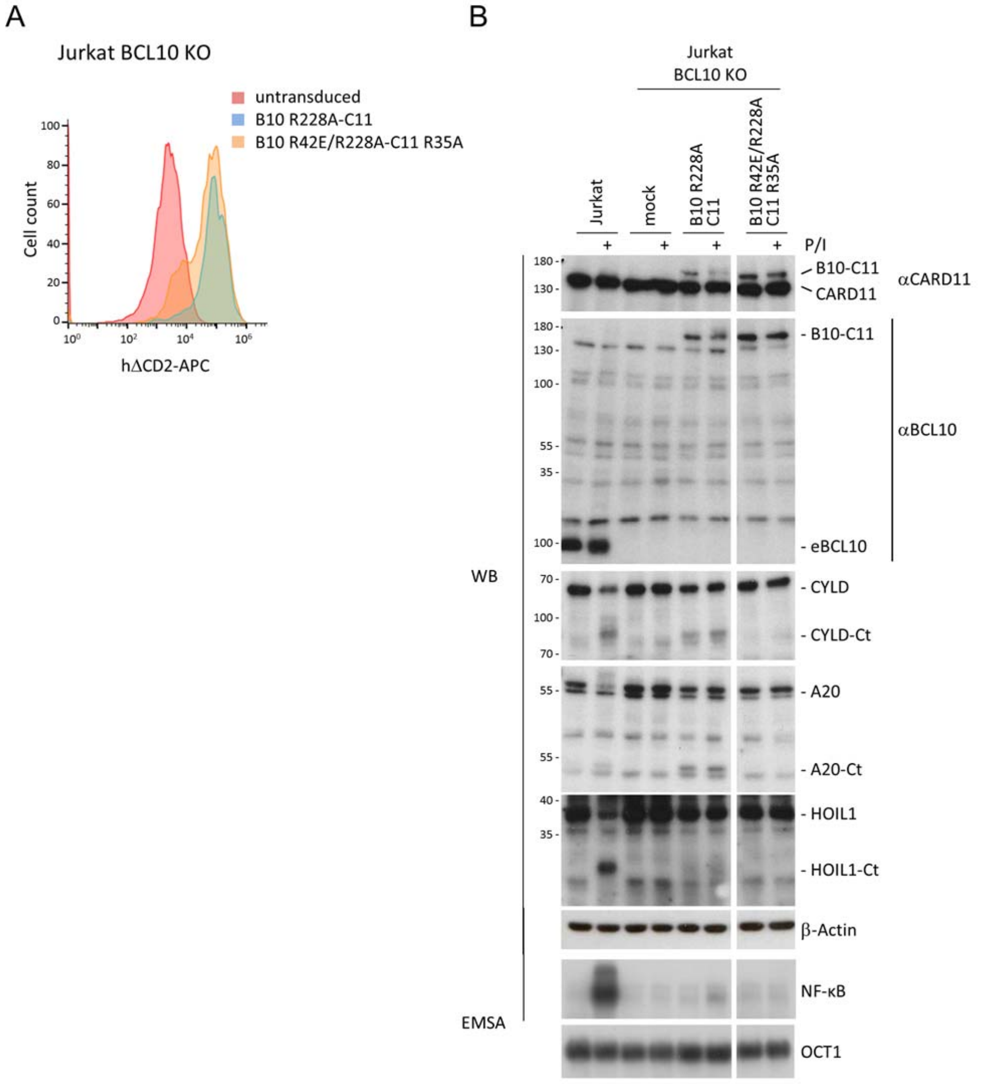

Supplementary Figure 4: Reconstitution of BCL10 KO Jurkat T cells with BCL10-CARD11 fusion proteins. (A) Transduction efficiency of BCL10 KO Jurkat T cells with BCL10-CARD11 (B10-C11) constructs determined by FACS using the surface marker ΔCD2. (B) Protein expression of the B10-C11 fusion constructs in BCL10 KO Jurkat T cells, cleavage of MALT1 substrates (CYLD, HOIL1, A20) and NF-κB activity (EMSA) was compared to mock and parental Jurkat T cells with or without P/I stimulation for 30 minutes.
